# First Preliminary Molecular Assessment of Ants from Cabo Verde

**DOI:** 10.3390/genes16070725

**Published:** 2025-06-22

**Authors:** Michael Joseph Jowers, Franco Guouman Ferreyra, Stephane Caut, José Carlos Brito, Raquel Vasconcelos

**Affiliations:** 1 Departamento de Biología y Geología, Física y Química Inorgánica, Universidad Rey Juan Carlos, 28933 Madrid, Spain; 2 Departamento de Zoología, Facultad de Ciencias, Universidad de Granada, 18071 Granada, Spain; francoguouman01@gmail.com; 3 National Institute of Ecology, 1210, Geumgang-ro, Maseo-myeon, Seocheon-gun 33657, Republic of Korea; 4 ANIMAVEG Conservation, 58 Avenue Allende, F-94800 Villejuif, France; 5 CIBIO, Centro de Investigação em Biodiversidade e Recursos Genéticos, InBIO Laboratório Associado, Campus de Vairão, Universidade do Porto, 4485-661 Vairão, Portugal; jcbrito@cibio.up.pt (J.C.B.); raquel.vasconcelos@uc.pt (R.V.); 6 BIOPOLIS Program in Genomics, Biodiversity and Land Planning, CIBIO, Campus de Vairão, 4485-661 Vairão, Portugal; 7 Departamento de Biologia, Faculdade de Ciências da Universidade do Porto, 4169-007 Porto, Portugal; 8 Department of Life Sciences, University of Coimbra, Calçada Martim de Freitas, 3004-504 Coimbra, Portugal

**Keywords:** island colonization, diversity, invasive species, Formicidae, Hymenoptera

## Abstract

Background/Objectives: Ants are one of the most abundant animal groups on the planet and have a considerable impact on ecosystems. In the Cabo Verde Archipelago, the study of invertebrates is very scarce and ants are no exception. Methods: In this work we focus on the taxonomic analysis of formicids and study their distribution and the possible presence of invasive species in the Cabo Verde Islands. In addition, the diversity of Cabo Verde ants is compared with that of the closest African coastal countries, Senegal and Mauritania, to study a possible colonization of African ants into the archipelago. For this, we use two molecular markers, cytochrome oxidase I and the wingless gene, to perform phylogenetic analyses and haplotype networks that facilitate identification. Results: Nine taxa were identified, five invasive species, *Paratrechina longicornis*, *Pheidole megacephala*, *Trichomyrmex destructor*, *Brachyponera sennaarensis*, and *Solenopsis globularia*, one endemic *Monomorium subopacum* and three unidentified species of native genera, *Monomorium* sp., *Lepisiota* sp. *Camponotus* sp. Conclusions: Molecular network patterns as well as phylogenetic analyses suggest that ants are widespread throughout the archipelago, a likely consequence of human introductions.

## 1. Introduction

**Figure 1:**
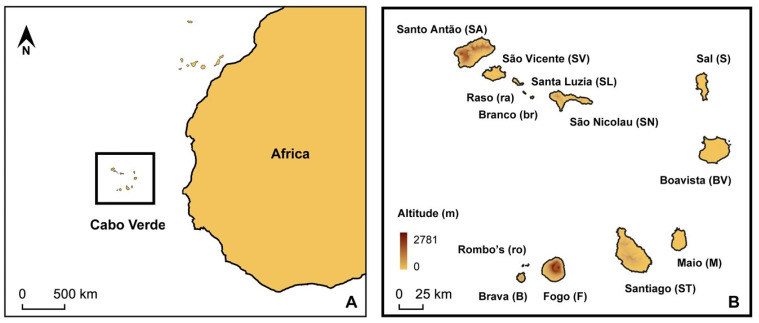
Location of the study area. (**A**) The map shows the Cabo Verde Islands in relation to Western Africa. (**B**) The image shows the Cabo Verde Islands and their names.

Ants (Hymenoptera: Formicidae) are one of the most abundant insects in the world and have an almost worldwide distribution, having colonized practically all possible terrestrial ecosystems [1]. Furthermore, they are of vital importance to the ecosystems in which they live, as they actively interact with other animals and plants, including humans [2]. Despite this, there are very limited studies of Hymenoptera in some areas, including some within biodiversity hotspots, such as the Cabo Verde Archipelago (Figure 1). However, they have been studied in some inventories carried out on the islands, such as in [3,4]. Currently, 39 different species of ants are recognized in Cabo Verde [4]. Most of the non-endemic native species have an Afrotropical origin, and it can be deduced that most of the biodiversity of formicids comes in some way from the Sahel region closest to the archipelago, in this case Mauritania and Senegal, aided by wind. It should be noted that many of the species from this region are considered invasive in many countries, so it is still under discussion whether they arrived naturally or have been introduced.

The Cabo Verde Archipelago is extremely arid and baron with scorching heat throughout the year. It barely rains and when it does it is often torrential and vegetation grows thereafter. The ants are often found in dry soil and are often encountered near human settlements. Of the 39 species 14 species were only encountered once or twice, which suggests species that have gone unreported [4]. Only three species are endemic (*Camponotus occasus*, *Cadiocondyla* sp. and *Monomorium boltoni*) but other unidentified species are likely endemic candidates too. Additionally, 24 species a native to the Afrotropical or Paleartic realms (i.e., *Brachyponera sennaarensis*). Nine of these species are tramp ants which have been widely spread through human commerce (e.g., *Pheidole megacephala* and *Trichomyrmex destructor*) but it is unknown if there are native or introduced in Cabo Verde. Two species, *Monomorium bicolor* and *Nylanderia jaegerskioeldi*, are not considered tramp ants are present and are likely introduced and seem to be common although restricted to Santiago. Seven species originate from the Indomalay or Australasian realms (e.g., *Paratrechina longicornis*, *Tapinoma melanocephalum*) and two further species (*Solenopsis globularia* and *Brachymyrmex cordemoyi*) are considered exotic New World ants. Human-induced environmental degradation and desertification and the negative effects of introduced plants and animals are the possible reason for the extremely low number of endemics in the archipelago [4]. Tramp ants are likely having detrimental ecological effects in Cabo Verde and causing extinction of other ant species there. An example is the likely extinction of the endemic *M. boltoni*, only known from Monte Gordo, the highest mountain of São Nicolau, by the most common ant in Cabo Verde, the invasive ante *P. megacephala*. Similarly, *T. destructor* is known from human settlements in Cabo Verde and is well known for its aggressive behavior. Thus, the spread of this species will have detrimental effects to the local fauna. Increasing efforts of exotic trees to counteract environmental degradation has likely increase the arrival of exotic ants [4].

How ants colonize islands is also central to understand patterns of natural versus introduced events. Ants colonize oceanic islands in three different ways: by flight, rafting, and dispersing with human help. Regarding the first way, winged reproductive ants are capable of traveling long distances in search of a mate, although the distance traveled differs greatly between species, ranging from 30 m for *Pheidole minutula* to 30 km for the fire ant *Solenopsis invicta* [5,6,7]. Despite being considerably long distances, it is not enough to reach the Cabo Verde Archipelago. However, the sandstorms that periodically affect the islands from continental Africa act as a driver of colonization, since they can reach speeds of 40 km/h and most of the islands [8]. Ants arriving by rafting are not common and this way is usually limited to islands near the mouths of very fast-flowing rivers and of an arboreal nature [9]. In this case, the ants arrive on logs or across the sea surface. The third way is, along with the first, the most common; boats or planes that arrive at the islands, whether transporting people or materials, can introduce ants to the archipelago [9]. This introduction of ants to Cabo Verde leads to the arrival of exotic species of formicids that may pose a danger to native and endemic ones, as some are considered invasive in other regions of the world [10]. A clear example is the longhorn crazy ant *P. longicornis*, one of the most abundant in the country, and considered invasive in practically all tropical and subtropical regions of the world, but still unknown whether it is native or exotic on the islands [4].

Invasive ant species usually have several queens (polygyny) in several nests (polydomy) and newly mated queens disperse on foot surrounded by workers from the same nest [11]. This behaviour means that their dispersal distance is not high, in response to this, they generate a continuous network of nests, so their density increases exponentially, which they maintain for long periods of time. Over time, invasive species greatly outnumber native ones, putting them at a disadvantage in the competition for resources, food, and habitat [11]. In addition, invasive species are extremely competitive, unlike native species, which tend to be more passive [11]. Many invasive species are major ecosystem modifiers, such as the Argentine ant, *Linepithema humile*, which harms several species of tree-dwelling ants by decreasing their foraging activity [12]. This results in a decrease in the diversity of native species, however, there are cases where the arrival of the invasive species creates new niches that can be occupied by the species already present or these native species that are ecologically similar and competitive enough to be able to cope with the invasive species [13]. It is also possible that the native species does not encounter the invasive species as it is not distributed in the same way and is therefore not affected by it, although this situation is not common due to the generalist nature of the exotic species [10,14,15,16]. Thus, conducting a new census with molecular markers of the formicids in Cabo Verde is essential to understand the real diversity throughout and help in the distinction between native and exotic species.

To this point, opportunistic sampling was performed to conduct molecular sequencing of the mitochondrial gene COI, and the nuclear gene Wingless (Wg). The main objective was to list the diversity of Cabo Verdean formicids and study their colonization patterns. More specifically, the aim was to: (1) molecularly identify the sequenced species, (2) identify their phylogenetic patterns, (3) evaluate their colonization patterns within the islands and in respect to the African coast, (4) detect the presence of invasive species.

## 2. Materials and Methods

Ant workers were collected from different regions in some of the Cabo Verde Islands (Santiago, Fogo, Rombo’s, Santa Luzia, Santo Antão, São Vicente, Branco, São Nicolau and Maio), as well as a few ant samples from Mauritania. This was an opportunistic sampling carried out while a series of other inventories, with more systematic sampling on Santa Luzia. Samples were collected mostly by visual surveys and with pitfalls. Samples were transported to NIE (South Korea) for molecular analyses and sequencing in absolute alcohol at room temperature. For the DNA extraction only one or two legs per ant (depending on the size of the ant) were used and thoroughly cut with a disposable scalpel blades and petri dishes for each sample, until the legs were completely grounded. By using legs there is a much less risk of bacterial or other contamination than if extracted from the digestive track such as from the abdomen or the head (i.e., mouth). Photos of the specimens were taken for posterior morphological identification. DNA extractions (DNA concentration; 20–40 ng/µL) were performed using a Qiagen DNeasy blood and tissue kit (Qiagen, Hilden, Germany) following the manufacturer’s instructions. The mitochondrial genes Cytochrome Oxydase I (COI) as well as the nuclear gene Wingless (Wg) were amplified by PCR. These gene fragments are highly informative in arthropod studies [17,18,19]. Primers LCO (L) 5′-GGTCAACAAATCATAAAGATATTGG-3′ and HCO (R) 5′ TAAACTTCAGGGTGACCAAAAAATCA-3′ [20] amplified a fragment of ~900 base pairs (bp) while primers Wg578F 5′-TGCACNGTGAARACYTGCTGGATGCG-3′ [21] and 1032R 5′-ACYTCGCAGCACCARTGGAA-3′ [22] amplified a fragment of ~480 bp. To avoid possible contamination, a positive and negative sample was loaded to the 1% agarose gel. Similarly, and to avoid unspecific DNA amplification we used an annealing temperature of 55 degrees to make the ant amplification more specific. All PCR products were sequenced in the forward and reverse directions with the same primers used for amplification. Complementary reads were used to resolve cases of ambiguous bases in Sequencer v5.4.6 (Gene Codes Corporation, Ann Arbor, MI, USA). The length of the alignments was of 641 bp for COI and 377bp for Wg. However, not all individuals had the same length in some alignments. Alignments were performed in SeaView [23]. Sequence data from this study were uploaded to GenBank (Supplementary Material Table S1).

For the phylogenetic trees, sequence GenBank blast were conducted to identify the most similar sequences to include in alignments (Supplementary Material Table S1). When GenBank species matches were equal or higher than 99% no phylogenetic trees were built. In the event that this percentage of similarity was less than 99%, a tree was created by selecting the closest species, choosing only one individual of each species and an outgroup chosen with respect to phylogenetic studies. Gene fragments (COI and Wg) were not concatenated as many samples from GenBank did not have both gene fragments available. All phylogenetic analyses were performed in the CIPRES platform [24]. For the *Camponotus* sp. phylogenetic tree, we selected the species *Myrmoteras cuneonodum* as an outgroup [25]. For the *Lepisiota* sp. phylogenetic trees, *Brachymyrmex depilis* was used as an outgroup [25], and *Adelomyrmex* sp. was used as an outgroup for the genus *Monomorium* [25]. In this case, all *Monomorium* species were included in the same phylogentic trees. Lastly, a phylogenetic tree (COI) was built to try to clarify the phylogenetic position and to assess *Solenopsis* sp. identity using *Chelaner antarcticum* as an outgroup [26].

Phylogenetic tree reconstructions for the non-concatenated gene fragments were performed using maximum likelihood (ML) and Bayesian inference (BI), using RAxML BlackBox v8.2.12 [27] and MrBayes v.3.2.7 [28], respectively. Only nodes with ML bootstraps and Bayesian posterior probabilities ≥ 75 are shown in the phylogenetic analyses. JModelTest2 v2.1.6 [29,30] was used to select the best fit evolutionary models. The chosen models for the phylogenetic analyses depended on the datasets (Wg trees: *Lepisiota* TIM2 + I and *Monomorium* TPM1uf + G; COI trees: *Lepisiota*, *Camponotus*, *Monomorium* TIM2 + I + G, and *Solenopsis* GTR + I + G). RAxML was used to run phylogenetic relationships with automatic run bootstraps and a codon partitioning model was used with estimated proportion of invariant sites (GTRGAMMA). MrBayes was run with codon partition and a two independent runs (each with four Markov chains) for 5,000,000 generations. Trees and parameters were sampled every 1000 generations. A majority-rule consensus tree was estimated by combining results from duplicated analyses, after discarding 25% of the total samples as burn-in. Trees were visualized and edited in Figtree v.1.4.4 [31], and later prepared as a graphic with Inkscape v.1.0.1 (http://www.inkscape.org accessed on 1 January 2023).

For the haplotype networks, only individuals with a similarity percentage greater than 95% were selected, except in cases where there was no sufficient species similarity and so the most similar sequences were selected. DNAsp V.6.12.3 [32] was used to create these networks, to assess polymorphic sites and to diphase ambiguous nuclear substitutions. PopART [33] was used to build the haplotype networks using Median joining networks [34]. Uncorrected *p*-distances were computed with 95% coverage partial deletion in MEGA X [35] (Supplementary Material Table S2).

## 3. Results

Nine different taxa were genetically identified through COI and Wg molecular markers: *B. sennaarensis*, *P. longicornis*, *P. megacephala*, *T. destructor*, *S. globularia*, *Camponotus* sp., *Lepisiota* sp., *Monomorium* sp., *M. subopacum*.

### 3.1. The Samsum Ant Brachyponera sennaarensis (Mayr, 1862)

**Figure 2:**
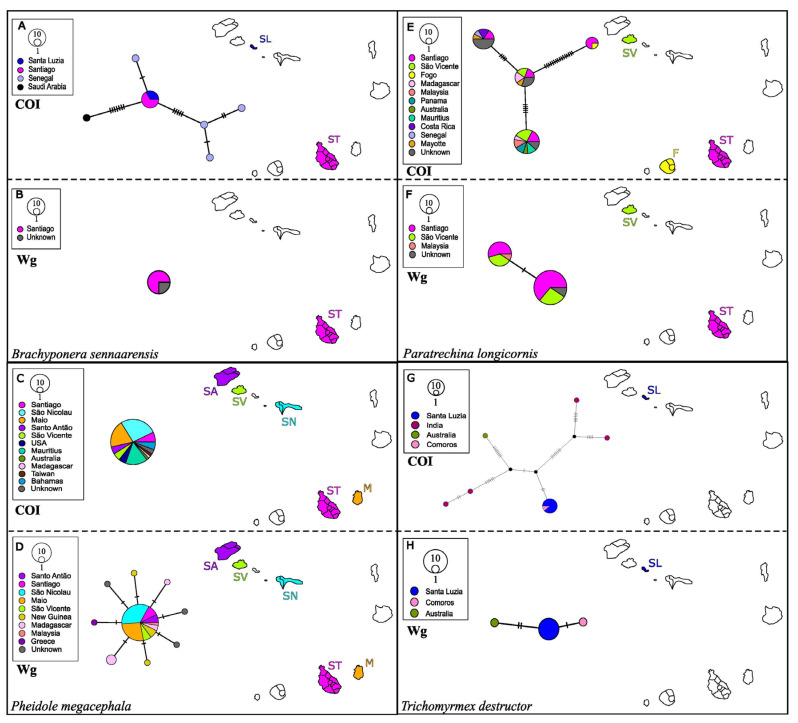
Haplotype networks of: *Brachyponera sennaarensis*: COI (**A**), Wg (**B**), *Pheidole megacephala*: COI (**C**), Wg (**D**), *Paratrechina longicornis* COI (**E**), Wg (**F**), and *Trichomyrmex destructor*: COI (**G**) and Wg (**H**).

In this case, it was not necessary to create phylogenetic trees since the similarity index of the GenBank sequences was higher than 99%, both in the mitochondrial gene (99.84%) and in the nuclear gene (100%). The closest match for the COI was *Pachycondyla* sp. and *P. sennaarensis* for Wg as *Brachyponera* was previously considered *Pachycondyla* [36]. This species has been previously recorded in all the islands of the Cabo Verde Archipelago [4]. *B. senaarensis* recovered the same haplotype from Santiago and Santa Luzia, and with close genetic affinity to Senegal, the geographically closest African country to the archipelago (Figure 2). Morphological identification confirmed that our specimens were indeed *B. sennaarensis* and not *Pachycondyla* sp.

### 3.2. The Longhorn Crazy Ant Paratrechina longicornis (Latreille, 1802)

**Figure 3:**
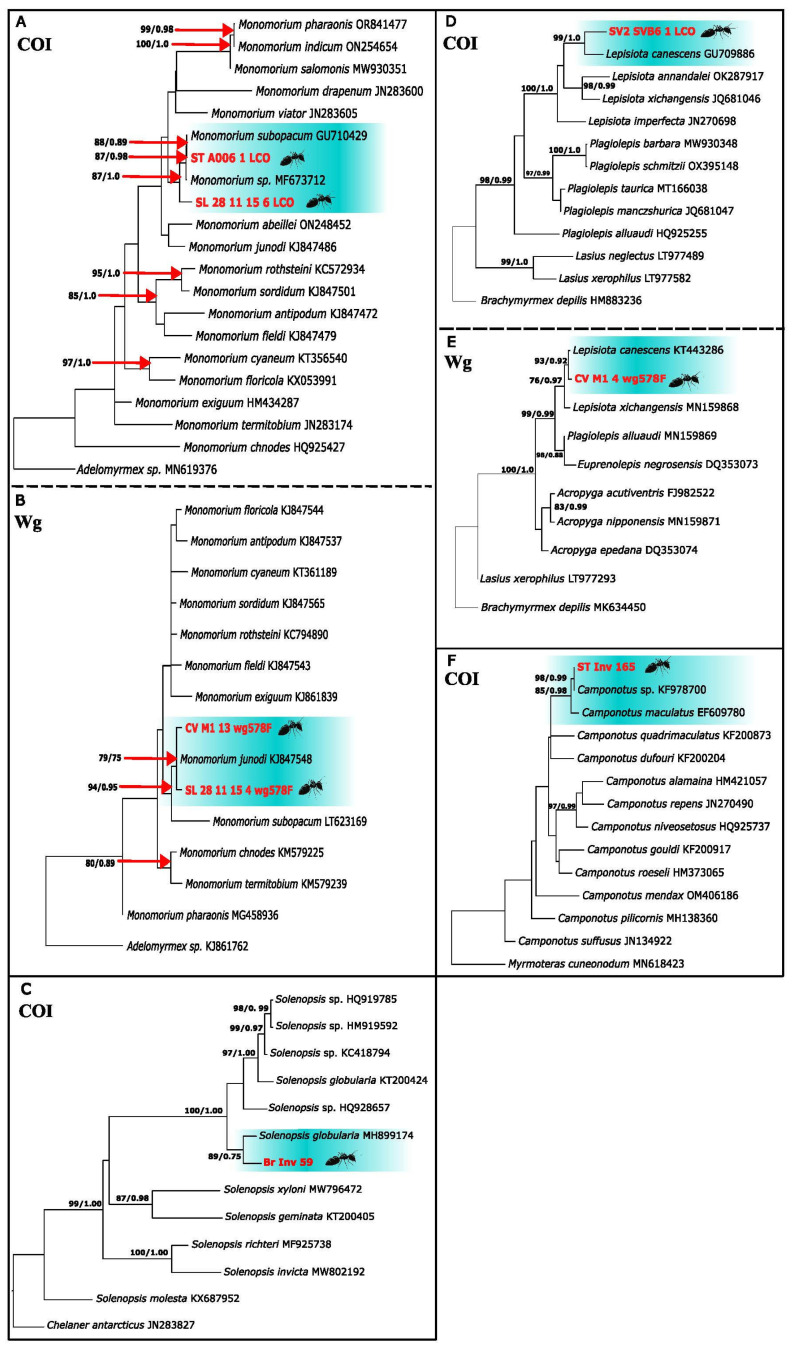
Maximum Likelihood best trees with bootstraps for the ML and from posterior probabilities for the Bayesian Inference for *Monomorium* sp.: COI (**A**), Wg (**B**), *Solenopsis globularia:* COI (**C**), *Lepisiota* sp.: COI (**D**), Wg (**E**) and *Camponotus* sp.: COI (**F**).

GenBank blasts resulted in a 100% match with *P. longicornis*, for both genes. This species has long been reported from Cabo Verde [3] and it is considered an invasive species. Figure 3 shows the same haplotype recovered from different regions worldwide, including Cabo Verde. *P. longicornis* is found on Fogo, Santiago, Santo Antão and São Vicente [3,4]. Of the four COI haplotypes, one of them is shared by Fogo and Santiago, and another two by Santiago and São Vicente. In addition, the Cabo Verdean individuals have exactly the same haplotype as individuals collected in different parts of the world, such as Senegal, Malaysia or Madagascar (Figure 2).

### 3.3. The African Big-Headed Ant Pheidole megacephala Fabricius, 1793

**Figure 4:**
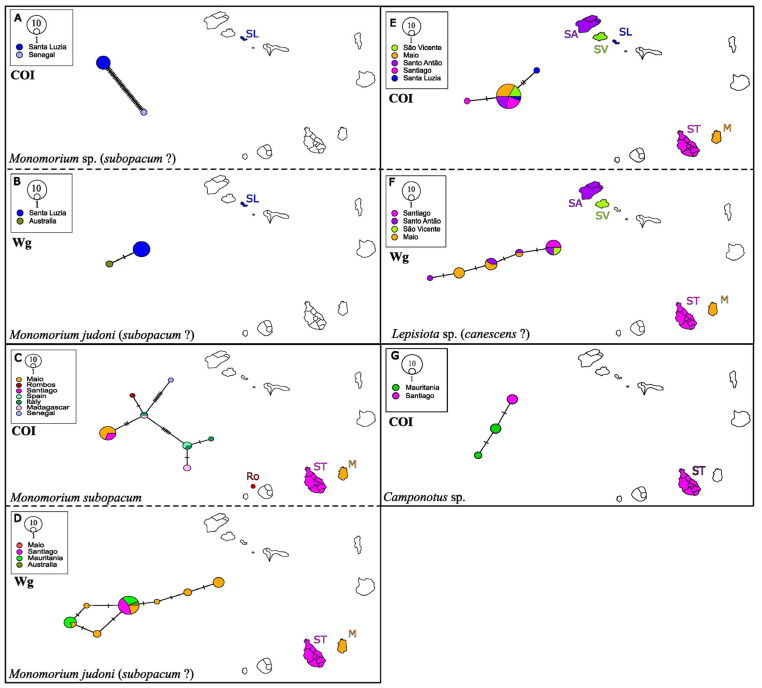
Haplotype networks of *Monomorium* sp.: COI (**A**,**C**), Wg (**B**,**D**), *Lepisiota* sp.: COI (**E**), Wg (**F**) and *Camponotus* sp.: COI (**G**).

Sequences match 100% with *P. megacephala*. This is an invasive species, also recorded in all the Cabo Verde Islands [4]. Figure 4 shows the same haplotype in different regions of the planet. *P. megacephala* from the archipelago recovered a single haplotype for both genes, where the samples from Santiago, Santo Antão, São Vicente, São Nicolau and Maio were grouped with individuals from very far regions, such as the United States of America or Australia (Figure 2). Samples from Santo Antão were morphologically identified as *P. megacephala*.

### 3.4. The Singapore Ant Trichomyrmex destructor (Jerdon, 1851)

The COI GenBank blasts match *T. destructor* (=*Monomorium*) 100%, while the Wg matched the same species at 99.73%. In Cabo Verde, it has been recorded on all islands except for Boavista, Maio and Sal [4]. It is an invasive species, however, unlike others studied in this work, in the COI haplotype network (Figure 2) only one haplotype is observed, shared between Santa Luzia and Comoros. The nuclear haplotype of the Cabo Verde sample only differs in two to three substitutions from samples from Australia and Comoros (Figure 2).

### 3.5. The Carpenter Ant Camponotus sp. Mayr, 1861

The closest COI match (98.74%), matched *Camponotus* sp. The next closest GenBank sample to those from Cabo Verde belonged to *C. maculatus*, however, its percentage of similarity is only 91.52%, so its species identity cannot be specified exactly. Therefore, a phylogenetic tree was built (Figure 3) to elucidate which species it could belong to by phylogenetic similarity.

As expected, the samples from this study (marked in red), which are represented by only one sequence, as they are practically identical, appear grouped together with *Camponotus* sp. with high support, which in turn form a clade with *C. maculatus*, also strongly supported. The fact that *Camponotus* sp. is identified only at the genus level is problematic for identification purposes. Nevertheless, our samples from Santiago were morphologically identified as *C. maculatus*. Intraspecific distances (Supplementary Material Table S2), show that the sequence that differs the least from that of Cabo Verde is *Camponotus* sp. with a distance of 1.08%, confirming that it is in fact similar. *C. maculatus* is the second most similar sequence with 9.01% of distance. *Camponotus* sp. was found on the island of Santiago and has a very close haplotype to individuals collected in Mauritania, a country located just above Senegal, not far from Cabo Verde. This species was previously recorded and considered to be native in São Nicolau [3] (Figure 4).

### 3.6. The Plagiolepidine Ant Lepisiota sp. Santschi, 1926

The closest COI GenBank match is *L. canescens* with a similarity of 90.73% (COI) and 98.11% (Wg). Due to the low COI percentage similarity, a phylogenetic tree was created to identify the species through phylogenetic relationships (Figure 4). In both phylogenetic trees, the samples from this study (marked in red) are grouped with *L. canescens* with high support in the COI (A) tree and 93% (ML) and Bayesian Posterior Probability (BPP) of 0.92 in the Wg (B) tree. In the interspecific distances (Supplementary Material Table S2), *L. canescens* appears in both the COI (A) and Wg (B) as the closest taxon with a divergence of 9.64% and 0.67%, respectively. This Afrotropical species has been recorded from Santiago, Santo Antão, Fogo, Maio, São Vicente, Sal and Brava [4]. The most frequent COI haplotype is from São Vicente, Maio, Santiago, Santo Antão and Santa Luzia, while Santiago, Santo Antão and São Vicente have the same Wg haplotype. Santo Antão and Maio constitute another two of the five haplotypes in both markers. Other haplotypes are recovered from Santa Luzia and Santiago (COI), and Santo Antão, and Maio (Wg) (Figure 3). Some of our samples from Santiago (ST_INV_168/ 178), São Vicente (SV_INV_149), Santa Luzia (SL_INV_211) and Fogo (F_INV_195) were morphologically identified as *Lepisiota cf canescens*.

### 3.7. The Pharaoh Ants Monomorium sp. And Monomorium subopacum (F. Smith, 1858)

The COI sequences from Santa Luzia recovered two highly divergent *Monomorium* and were therefore separated into two haplotype networks (per gene, Figure 4) from those taken from the rest of the islands. The Santa Luzia closest GenBank match was to *Monomorium* sp. with 99.46% similarity, and the remaining *Monomorium* sequences from the other islands matched *M. subopacum*, with 99.47%. The Wg (B) sequences did not recover two highly divergent lineages, but recovered one, and all matched *M. junodi* with 99.72% similarity. This difference between the mitochondrial and nuclear genes and the high divergence of the Santa Luzia samples leads us to build a phylogenetic tree to resolve its phylogenetic position.

In the COI tree (Figure 3), the samples from Cabo Verde were grouped with *Monomorium* sp. (bootstrap of 87% and BPP:0.98) and with its sister species *M. subopacum* with a bootstrap of 88% and BPP of 0.89, respectively. In the Wg (Figure 4), the clade that groups the sequences from the archipelago with *M. junodi* was weakly supported. In the interspecific distances (Supplementary Material Table S2), for the COI data, the difference between the two Cabo Verde sequences is 7.35%, with the closest sequence to Santa Luzia ants being *Monomorium* sp. with 1.77% and *M. subopacum* to the rest of the sequences from other islands with 0.53%. In the Wg gene, the samples from Santa Luzia and the rest of the islands differ by 0.64%, with the closest sequence to both being *M. junodi* with 0.32%. It should be noted that the rest of the species do not differ too much, all below 3%. Morphological identification was also inconclusive.

In the COI dataset, the samples from Santa Luzia recovered a single haplotype that is very divergent and differentiated from the one from Santiago. The sample from Rombo’s recovered a single haplotype that differs by six mutational steps from the Maio and Santiago haplotype. Both the sequences from Rombo’s and those from Maio and Santiago are close to the haplotype of individuals from Italy and Madagascar. In the Wg network, the sequence from Maio and Santiago form a haplotype with samples from Australia and Mauritania, and the Santa Luzia individuals are also very divergent and clustering with Australian individuals, with two mutational steps of difference between them (Figure 3).

### 3.8. The Globular Thief Ant Solenopsis globularia Westwood, 1840

*S. globularia* was the closest species in GenBank (COI), with 96.89% similarity. Although this percentage is not very low, it is not high enough to ascertain its identity. The individual from Cabo Verde (marked in red) grouped with *S. globularia* with high support. In the interspecific distances, the closest sequence to that from Cabo Verde is *S. globularia* with a 2.68% genetic difference. The rest of the species are very distant with more than 16% distance (Supplementary Material Table S2). This is the only species that was found on Branco Islet. Two species were described by [3] (Figure 3).

## 4. Discussion

### 4.1. Species Identification and Biodiversity

Overall, nine different taxa were genetically identified: *B. sennaarensis*, *P. longicornis*, *P. megacephala*, *T. destructor*, *S. globularia*, *M. subopacum*, *Camponotus* sp., *Lepisiota* sp., *Monomorium* sp., and. Despite the opportunistic sampling of this study, analyzing the genetic diversity present in the Cabo Verde populations and comparing them with genetic databases is an important first step to assess the archipelago’s native ant biodiversity [37]. In addition, individuals from poorly studied uninhabited islands such as Santa Luzia and Branco are of great interest for the study of ant biodiversity. For example, on Santa Luzia a group of individuals of the genus *Monomorium* was highly divergent to the rest of the islands, and on Branco, an individual of the genus *Solenopsis* did not match any species present in GenBank from Cabo Verde. Finally, the sequencing of the individuals collected in this study will serve as a reference for future studies on the biodiversity or taxonomy in the islands. Although some of the taxa were easier to identify through GenBank blasts (*B. sennaarensis*, *P. longicornis*, *P. megacephala* and *T. destructor*), for many of them (*Camponotus* sp., *Lepisiota* sp., *Monomorium* sp. *M. subopacum* and *S. globularia*) it was necessary to establish phylogenetic relationships to infer their taxonomy.

Regarding *Camponotus* sp., three different species have been described in Cabo Verde: *C. maculatus*, *C. occasus* and *C. guttatus* [3,4]. *C. maculatus* is a species native to Africa that is found on most of the Cabo Verde Islands, except for Santo Antão, Sal, São Nicolau and São Vicente. *C. occasus* is a species endemic to the island of Santo Antão, so we can rule out that it is this species since the individual in this study was collected on the island of Santiago. *C. guttatus* is a species native to Africa that has been recorded on the islands of Santiago and Maio. There is a fourth species that has been described on the islands, *C. foraminosus* (https://www.antwiki.org/wiki/Camponotus_foraminosus, accessed 1 June 2024). This species has also been recorded in Ghana, the country from where the individual from GenBank with the closest match to our sequence was collected. This sequence appeared in the tree as a sister species, however, there are few records of this species in Cabo Verde and they date from the years 1879 and 1910 [38,39], probably misidentifications, as further species lists of Cabo Verde exclude them [3,4]. The GenBank *Camponotus* sp. sequence is from Ghana, and the Cabo Verde individual was collected on Santiago Island, where this species has been previously described. In addition, it has also been described in regions close to Mauritania. Therefore, all three species (*C. maculatus*, *C. guttatus* or *C. foraminosus*) are possible candidates. All the species named above are not registered in GenBank.

*L. canescens*, an invasive species, has been recorded in all Cabo Verde Islands except on São Nicolau and Boavista [4]. Initially, individuals found in Cabo Verde were identified as *L. capensis* (see [3]), however, ref. [4] concluded that these were identification errors and that they were, in fact, individuals of *L. canescens*. On the other hand, even though the intraspecific genetic distances with respect to the nuclear genes are low, the COI is very divergent. Taking this into account, and that there are no records of *L. capensis* in GenBank we cannot be sure of the taxonomic identification of individuals of this genus until sequences of this invasive species are available. If they are *L. canescens*, it would be the first time that the species would be recorded on the island of Santo Antão. Alternatively, it could be a new or undescribed lineage or species for the islands.

In the case of *Monomorium* sp., it is not possible to specify which species the Santa Luzia sequences correspond to, since although the interspecific distance with *M. junodi* is low, the Wg phylogenetic tree is not very well supported and COI distances are very different. As for *M. subopacum*, although they are grouped with a very high bootstrap in both trees, the interspecific distances of the studied specimens to that taxa are very high for both markers. The samples from the rest of the islands are possibly *M. subopacum* since in both trees they are grouped in the same clade and their distances are low for both markers. In fact, the *p*-distances between the *M. subopacum* and *M.* sp GenBank samples have a low divergence of 1.59%, which suggests that they are likely the same species (Supplementary Material Table S2). Furthermore, unlike *M. junodi*, *M. subopacum* has been previously described in Cabo Verde [4].

Finally, the following *Solenopsis* species have been recorded in the Cabo Verde Archipelago: *S. globularia*, *S. orbuloides*, *S. orbula* and an undescribed species. The collected specimen is most likely *S. globularia*, since the interspecific genetic distances to that taxa are low (COI; 2.68%) and it is also found on all the Cabo Verde Islands. This native species is found on all the islands except Fogo. *S. orbula* is a species that has only been found on Santo Antão, although these might be a misidentification of existing records according to [4]. The undescribed taxon was recorded on São Nicolau, from where it is presumed to be endemic. Finally, there are not many records of ants on Branco Islet, therefore the diversity of this group on this area of the archipelago remains unknown [4].

### 4.2. Cabo Verde Archipelago Colonization and Dispersal

Shared haplotypes between islands suggest good connections between inhabited islands, for example there is a total of nine ports spread across the island of São Vicente, Santiago, Sal, Santo Antão, Boavista, Fogo, Maio, Brava and São Nicolau. Of all of them, only the ports of São Vicente, Santiago and Sal are international. Therefore, individuals from these islands may have very similar haplotypes resulting from the constant maritime traffic among them.

The island of Santa Luzia is accessed mostly by fishing boats from São Vicente, therefore the divergence found in *Monomorium* sp. probably relates to the fact that it is relatively isolated from the rest of the islands. Sequences from Santa Luzia that share a haplotype with other islands are likely introductions from São Vicente fishing vessels. On the other hand, *T. destructor*, which has only been collected on Santa Luzia with a same haplotype as Comoros, might have arrived on São Vicente with subsequent spread to Santa Luzia. A similar case occurs on Rombo’s, which is a group of islets located north of Brava Island, where *Monomorium* likely colonized the islets by fishing boats from Brava resulting in different haplotypes to those from Santiago and Maio.

The Cabo Verde Archipelago is located near the African continent, west of the coast of Senegal. As mentioned above, ants usually colonize oceanic islands mainly in two ways: through winged reproductives and through human intervention [9]. Both Senegal and Mauritania are the closest countries to Cabo Verde, so it would be expected that ants of the archipelago would resemble those of the African coast. Ants from oceanic islands usually resemble those of the nearest continent and in this case are likely aided by sandy winds from the Sahel. Of the sequences analysed, *B. sennaarensis* and *P. longicornis* shared or had high similarity to those from Senegal and *Camponotus* sp. recovered a haplotype very similar to sequences from Mauritania. In addition, *P. megacephala*, various species of *Camponotus* (including *C. foraminosus*) and species of the genus *Monomorium*, such as *M. subopacum* are also present in Senegal [40]. In Mauritania, ants considered invasive such as *P. megacephala* and *P. longicornis*, and other native species, such as *L. canescens* are present [41], as in this study. In addition, according to [4], many of the Cabo Verde species have an Afrotropical origin, including those mentioned above. In Cabo Verde, it is unknown whether they are native or introduced species, however, the haplotype networks of this study show how the same haplotype is present in many islands and in remote countries, suggesting introductions.

### 4.3. Invasive and Exotic Ants in Cabo Verde

Invasive ants represent both an economic and conservation problem by negatively affecting native and endemic species. In this study, a total of five species are considered invasive; *P. longicornis*, *P. megacephala*, *T. destructor*, *B. sennaarensis*, and *S. globularia*. These species exhibit aggressive behaviour, a great capacity for colonization and the displacement of native species and, in addition, they are widely distributed throughout the world and are considered invasive species in practically all countries, including Cabo Verde [10,11,15].

The crazy ant *P. longicornis* is the most widely distributed ant in the world and is generally found acting as a pest in gardens and houses [42]. It is scarce in intact environments and quite common in those disturbed by human activity [42]. Its cosmopolitan distribution is mainly due to its great ease in thriving in any human habitat, even on maritime vessels [43]. Although it is considered, like *B. sennaarensis*, a vagrant species, it can also act as an invasive species, displacing native species, as could be happening in Cabo Verde with the endemic species *M. boltoni* [4,42,44]. In fact, it is one of the most dispersed species around the world [4], which is reflected in the haplotype networks (Figure 2). Its origin is still under discussion, but [45] proposed an origin in India, since it is distributed throughout the country without interruption. On the other hand, ref. [46] proposed an African origin because the other species of the genus are restricted to the Afrotropical region.

The African big-headed ant *P. megacephala* is a species that acts with great aggressiveness towards native species both in its region of origin and in regions where it is considered invasive worldwide [47]. This causes a dominance over the rest of the species, especially on islands where the native species are very passive towards other colonies [48]. Their ability to invade other colonies causes the destabilization of the native species and has been observed to negatively affect other invertebrates as well [11,48,49,50].

The Singapore ant *T. destructor* is considered an invasive species that usually acts as a pest in arid regions disturbed by humans and inside houses in more humid regions [51]. On islands, *T. destructor* usually spreads easily and then rapidly reduces its population [44]. For example, in the Canary Islands the species has not been sighted since 1929, being relatively abundant in the past [52]. On the other hand, in Cabo Verde, the species is expanding [44]. This is a species that, like *P. longicornis* and *P. megacephala*, are capable of displacing native species, in addition to having a great destructive capacity [44,52]. The recovery of this species only on Saint Luzia and the Comoros may be due to the small number of records in GenBank for this species and samples for this study, as *T. destructor* was only found on the island of Santa Luzia.

The Globular Thief ant *S. globularia* is endemic to the new world and had spread to extremely isolated islands (e.g., Hawaii, French Polynesia, Philippines) as well as the western coast of Africa including Senegal. All populations of *S. globularia* outside the New World are probably exotic, introduced through human commerce [53]. On Cabo Verde it is extremely widespread on all nine inhabited islands, and is mostly found in gardens and in *Acacia* stands and it most likely colonizes new areas in potted plants [53].

Finally, the Asian needle ant *B. sennaarensis* is considered a tramp ant due to its expansion and distribution throughout the world due to human activity, but without having a negative impact on biodiversity [54]. All the invasive species in this study have in common the structure of haplotype networks, where the haplotypes present in the Cabo Verde Islands match haplotypes in dispersed regions of the planet. *B. sennaarensis* and *P. megacephala* are native to the Afrotropical region [47,48,55], so they could have colonized the islands naturally.

## 5. Conclusions

The lower formicid biodiversity (nine taxa), compared to the 39 species present in Cabo Verde [4], was expected due to the sampling protocol. Yet, this study revealed some novel insights to never sampled islands in the archipelago. For example, this study closes the knowledge gap in the desert islands (three uninhabited islands of Cabo Verde that are now nature reserves), a region that has remained unexplored for its ant biodiversity by previous studies [3,4]. In fact, Wetterer and Espadaler (2021) [4] addressed that future research conducted during the rainy season on Santa Luzia, Branco, and Raso, could be particularly significant ad they could be havens for endemic ant species. Although our study did not reveal endemic species, the presence of a *S. globularia* on Branco, is the first record at the island. In addition, *B. sennarensis*, *T. destructor*, *Lepisiota* sp., *M. subopacum*/sp. were all recovered from Saint luzia and are all therefore new records there. In addition, this study revealed the presence of *M. subopacum* for the first time on Rombo’s, an uninhabited archipelago and integral reverse area with no ant occurrences. Most importantly, this study reveals for the first time the colonization and genetic connectivity of some ants throughout the archipelago, a novel insight into formicids in Cabo Verde.

### Future Prospects

Opportunistic and uneven sampling, with the exception of Santa Luzia, is a limitation for a generalized biodiversity assessment of the archipelago. The collecting of multiple ants at particular sites inflated the haplotype size as the ants were likely from the same colonies. Morphological vouchers would additionally help for future inventories. In addition, except for Santa Luzia, Branco, and Rombo’s, the rest of the islands are inhabited and well connected by ports and airports, a likely introduction route to and between the islands. Despite some study limitations, these results show for the first time the molecular networks of nine species of ants and the phylogenetic relationships of several species throughout the archipelago. Carrying out an exhaustive inventory of the archipelago’s ants will be important to understand how invasive species might affect native species. In addition, the diversity of the nearest African coast must be taken into greater consideration in order to specify colonization patterns.

## Data Availability

The original data has been submitted to GenBank (accession numbers are pending).

## Supplementary Materials

The following supporting information can be downloaded at https://www.mdpi.com/article/10.3390/genes16070725/s1, Table S1: Sample ID codes and GenBank accessions from this study, GenBank Blasts according to COI and WG markers, and localities; Table S2: *P*-uncorrected distance matrices.

## References

[1] Kass J.M., Guénard B., Dudley K.L., Jenkins C.N., Azuma F., Fisher B., Parr C.L., Gigg H., Longino J.T., Ward P.S. (2022). The global distribution of known and undiscovered ant biodiversity. Sci. Adv..

[2] Ellison A.M., Gotelli N.J. (2021). Ants (Hymenoptera: Formicidae) and humans: From inspiration and metaphor to 21st-century symbiont. Myrmecol. News.

[3] Báez M., Oromí P., Arechavaleta M., Zurita N., Marrero M.C., Martín J.L. (2005). Arthropoda. Lista Preliminar de Especies Silvestres de Cabo Verde (Hongos, Plantas y Animales Terrestres).

[4] Wetterer J.K., Espadaler X. (2021). Ants (Hymenoptera: Formicidae) of the Cabo Verde Islands. Trans. Am. Entomol. Soc..

[5] Wojcik D.P. (1983). Comparison of the Ecology of Red Imported Fire Ants in North and South America. Fla. Entomol..

[6] Bruna E.M., Izzo T.J., Inouye B.D., Uriarte M., Vasconcelos H.L. (2011). Asymmetric dispersal and colonization success of Amazonian plant-ants queens. PLoS ONE.

[7] Helms J.A. (2017). The flight ecology of ants (Hymenoptera: Formicidae). Myrmecol. News.

[8] Gama C., Tchepel O., Baldasano J.M., Basart S., Ferreira J., Pio C., Cardoso J., Borrego C. (2015). Seasonal patterns of Saharan dust over Cape Verde—A combined approach using observations and modelling. Tellus B.

[9] Morrison L.W. (2016). The ecology of ants (Hymenoptera: Formicidae) on islands. Myrmecol. News.

[10] Wittman S.E. (2014). Impacts of invasive ants on native ant communities (Hymenoptera: Formicidae). Myrmecol. News.

[11] Holway D.A., Lach L., Suarez A.V., Tsutsui N.D., Case T.J. (2002). The causes and consequences of ants invasions. Annu. Rev. Ecol. Syst..

[12] Westermann F.L., Suckling D.M., Lester P.J. (2014). Disruption of Foraging by a Dominant Invasive Species to Decrease Its Competitive Ability. PLoS ONE.

[13] Shimoji H., Suwabe M., Kikuchi T., Ohnishi H., Tanaka H., Kawara K., Hidaka Y., Enoki T., Tsuji K. (2022). Resilience of native ant community against invasion of exotic ants after anthropogenic disturbances of forest habitats. Ecol. Evol..

[14] Zina V., Branco M., Franco J.C. (2020). Impact of the Invasive Argentine Ant in Citrus Agroecosystems: Effects on the Diversity and Frequency of Native Ant Species Foraging on Tree Canopy. Insects.

[15] Archury R., Holway D.A., Suarez A.V. (2021). Pervasive and persistent effects of ant invasion and fragmentation on native ant assemblages. Ecology.

[16] Siddiqui J.A., Bamisile B.S., Khan M.M., Islam W., Hafeez M., Bodlah I., Xu Y. (2021). Impact of invasive ant species on native fauna across similar habitats under global environmental changes. Environ. Sci. Pollut. Res. Int..

[17] Lucky A. (2011). Molecular phylogeny and biogeography of the spider ants, genus Leptomyrmex Mayr (Hymenoptera: Formicidae). Mol. Phylogenet. Evol..

[18] Schär S., Talavera G., Rana J.D., Espadaler X., Cover S.P., Shattuck S.O., Vila R. (2022). Integrative taxonomy reveals cryptic diversity in North American *Lasius* ants, and an overlooked introduced species. Sci. Rep..

[19] Pava-Ripoll M., Miller A.K., Ziobro G.C. (2023). Development of a Multiplex Polymerase Chain Reaction (PCR) Assay for the Potential Detection of Insect Contaminants in Food. J. Food Prot..

[20] Folmer O., Black M., Hoeh W., Lutz R., Vrijenhoek R. (1994). DNA primers for amplification of mitochondrial cytochrome c oxidase subunit I from diverse metazoan invertebrates. Mol. Mar. Biol. Biotechnol..

[21] Ward P.S., Brady S.G., Fisher B.L., Schultz T.R. (2015). The evolution of myrmicine ants: Phylogeny and biogeography of a hyperdiverse ant clade (Hymenoptera: Formicidae). Syst. Entomol..

[22] Abouheif E., Wray G.A. (2002). Evolution of the gene network underlying wing polyphenism in ants. Science.

[23] Gouy M., Guindon S., Gascuel O. (2010). SeaView version 4: A multiplatform graphical user interface for sequence alignment and phylogenetic tree building. Mol. Biol. Evol..

[24] Miller M.A., Pfeiffer W., Schwartz T. Creating the CIPRES Science Gateway for inference of large phylogenetic trees. Proceedings of the 2010 Gateway Computing Environments Workshop (GCE).

[25] Ward P.S., Blaimer B.B., Fisher B.L. (2016). A revised phylogenetic classification of the ant subfamily Formicinae (Hymenoptera: Formicidae), with resurrection of the genera Colobopsis and Dinomyrmex. Zootaxa.

[26] Ward P.S., Downie D.A. (2005). The ant subfamily Pseudomyrmecinae (Hymenoptera: Formicidae): Phylogeny and evolution of big-eyed arboreal ants. Syst. Entomol..

[27] Stamatakis A. (2014). RAxML Version 8: A tool for Phylogenetic Analysis and Post-Analysis of Large Phylogenies. Bioinformatics.

[28] Ronquist F., Teslenko M., van der Mark P., Ayres D.L., Darling A., Höhna S., Larget B., Liu L., Suchard M.A., Huelsenbeck J.P. (2012). MRBAYES 3.2: Efficient Bayesian phylogenetic inference and model selection across a large model space. Syst. Biol..

[29] Darriba D., Taboada G.L., Doallo R., Posada D. (2012). jModelTest 2: More models, new heuristics and parallel computing. Nat. Methods.

[30] Guindon S., Gascuel O. (2003). A simple, fast and accurate method to estimate large phylogenies by maximum-likelihood. Syst. Biol..

[31] Rambaut A. (2018). Figtree, Version 1.4.4..

[32] Rozas J., Ferrer-Mata A., Sánchez-DelBarrio J.C., Guirao-Rico S., Librado P., Ramos-Onsins S.E., Sánchez-Gracia A. (2017). DnaSP 6: DNA Sequence Polymorphism Analysis of Large Datasets. Mol. Biol. Evol..

[33] Leigh J.W., Bryant D. (2015). PopART: Full-feature software for haplotype network construction. Methods Ecol. Evol..

[34] Bandelt H., Forster P., Röhl A. (1999). Median-joining networks for inferring intraspecific phylogenies. Mol. Biol. Evol..

[35] Kumar S., Stecher G., Li M., Knyaz C., Tamura K. (2018). MEGA X: Molecular Evolutionary Genetics Analysis across Computing Platforms. Mol. Biol. Evol..

[36] Schmidt C.A., Shattuck S.O. (2014). The higher classification of the ant subfamily Ponerinae (Hymenoptera: Formicidae), with a review of ponerine ecology and behavior. Zootaxa.

[37] Coker O.M. (2017). Importance of genetics in conservation of biodiversity. Nig. J. Wildl. Manag..

[38] Forel A. (1879). Études myrmécologiques en 1879 (deuxième partie [1re partie en 1878]). Soc. Vaud. Sci. Nat..

[39] Forel A. (1910). Ameisen aus der Kolonie Erythräa. Gesammelt von Prof. Dr. K. Escherich (nebst einigen in West-Abessinien von Herrn A. Ilg gesammelten Ameisen). Zool. Jahrb. Abt. Syst. Geog. Biol. Tiere.

[40] Diame L., Taylor B., Blatrix R., Vayssières J.F., Rey J.Y., Grechi I., Diarra K. (2017). A preliminary checklist of the ant (Hymenoptera, Formicidae) fauna of Senegal. J. Insect Biodivers..

[41] Taheri A., Wetterer J.K., Najjari A., Reyes-López J.L. (2024). Checklist of the Ants of Mauritania (Hymenoptera: Formicidae). Trans. Am. Entomol. Soc..

[42] Wetterer J.K. (2008). Worldwide spread of the longhorn crazy ant, *Paratrechina longicornis* (Hymenoptera: Formicidae). Myrmecol. News.

[43] Harris R.J., Abbott K., Barton K., Berry J.A., Don W., Gunawardana D.N., Lester P.J., Rees J.S., Stanley M.C., Sutherland A. (2007). Invasive ant pest risk assessment project for Biosecurity New Zealand. N. Z. J. Zool..

[44] Wetterer J.K., Miller S.E., Wheeler D.E., Olson C.A., Polhemus D.A., Pitts M., Ashton I.W., Himler A.G., Yospin M.M., Helms K.R. (1999). Ecological dominance by *Paratrechina longicornis* (Hymenoptera: Formicidae), an invasive tramp ant, in Biosphere 2. Fla. Entomol..

[45] Wasmann E. (1905). Zur Lebensweise einiger in- und ausländischer Ameisengäste (148. Beitrag zur Kenntnis der Myrmecophilen und Termitophilen). Z. Wiss. Insektenbiol..

[46] LaPolla J.S., Fisher B.L. (2014). Then there were five: A reexamination of the ant genus *Paratrechina* (Hymenoptera, Formicidae). Zookeys.

[47] Dejean A., Moreau C.S., Kenne M., Leponce M. (2008). The raiding success of *Pheidole megacephala* on other ants in both its native and introduced ranges. Comptes Rendus Biol..

[48] Wetterer J.K. (2014). Worlwide spread of the African big-headed ant, *Pheidole megacephala* (Hymeno-ptera: Formicidae). Myrmecol. News.

[49] Dejean A., Moreau C.S., Uzac P., Le Breton J., Kenne M. (2007). The predatory behavior of *Pheidole megacephala*. Comptes Rendus Biol..

[50] Hoffmann B.D., Andersen A.N., Hill G. (1999). Impact of an introduced ant on native rainforest invertebrates: *Pheidole megacephala* in monsoonal Australia. Oecologia.

[51] Wetterer J.K. (2009). Worldwide spread of the destroyer ant, *Monomorium destructor* (Hymenoptera: Formicidae). Myrmecol. News.

[52] Espadaler X., Bernal V. (2003). Exotic ants in the Canary Islands (Hymenoptera, Formicidae). Vieraea.

[53] Wetterer J.K. (2019). Geographic Spread of *Solenopsis globularia* (Hymenoptera, Formicidae). Sociobiology.

[54] Wetterer J.K. (2013). Geographic spread of the samsum or sword ant, Pachycondyla (Brachyponera) sennaarensis (Hymenoptera: Formicidae). Myrmecol. News.

[55] Dejean A., Lachaud J.P. (1994). Ecology and behavior of the seed eating ponerine *Brachyponera sennaarensis* (Mayr). Insect Soc..

